# Recent Developments, Applications, and Future Prospects of Advanced Hearts-on-a-Chip

**DOI:** 10.3390/mi17091079

**Published:** 2026-09-13

**Authors:** Aerin Kang, Hayun Lee, Gurnho Park, Taehong Kim, Hyoju Kim, Timothy Park, Sydney Staley, Miriam Kang, Youngbok (Abraham) Kang

**Affiliations:** Department of Mechanical, Civil, and Biomedical Engineering, George Fox University, 414 N. Meridian Street, #6088, Newberg, OR 97132, USA; aerink1224@gmail.com (A.K.); 205hylee@gmail.com (H.L.); gurnhopark@gmail.com (G.P.); taehongkim001@gmail.com (T.K.); juliek080909@gmail.com (H.K.); timopark1525@gmail.com (T.P.); sstaley22@georgefox.edu (S.S.); mkang25@georgefox.edu (M.K.)

**Keywords:** heart-on-a-chip, heart model, cardiomyocytes, electrical stimulation, mechanical stimulation

## Abstract

Heart-on-a-chip (HoC) platforms represent a cutting-edge convergence of microfluidics and cellular biology, designed to imitate the complex physiological, electrical, and mechanical environments of a human heart. HoC devices are used to culture and observe cardiac cells in a highly customizable environment while allowing for real-time observations. However, current HoC systems face critical translational bottlenecks. Most notably, laboratory-grown cardiomyocytes consistently exhibit structural and functional immaturity and a lack of cellular complexity. This review evaluates recent engineering and biological strategies engineered to address these limitations. We dissect advancements in multicellular co-culture, biomimetic electrical/mechanical stimulation regimes, and microvascular fabrication techniques aimed at driving functional cell maturation. Finally, we highlight current cardiac model applications, existing challenges, and future perspectives.

## 1. Introduction

Cardiovascular disease (CVD), commonly referred to as heart disease, has surpassed cancer mortality rates and has become the leading cause of death worldwide [1]. To study this fatal and silent disease, researchers develop and utilize human clinical trials, animal research, and laboratory models. Clinical trials test new drugs and medical devices on voluntary patients by tracking their effectiveness and changes in health conditions over a long period. Clinical studies often require years to complete, and the majority of them fail due to the inadequacy of the drug or device candidates. In addition, experimenting on humans raises significant ethical concerns, so human rights are prioritized over scientific discoveries. As for animal models, researchers rely on animal testing for preclinical drug testing, disease modeling, and harvesting of sample cells. While animal testing minimizes the need for human trials, it cannot be a complete replacement due to the fundamental differences between animal and human physiology. Also, ethical concerns still surround experimenting on animals despite regulations.

**Figure 1:**
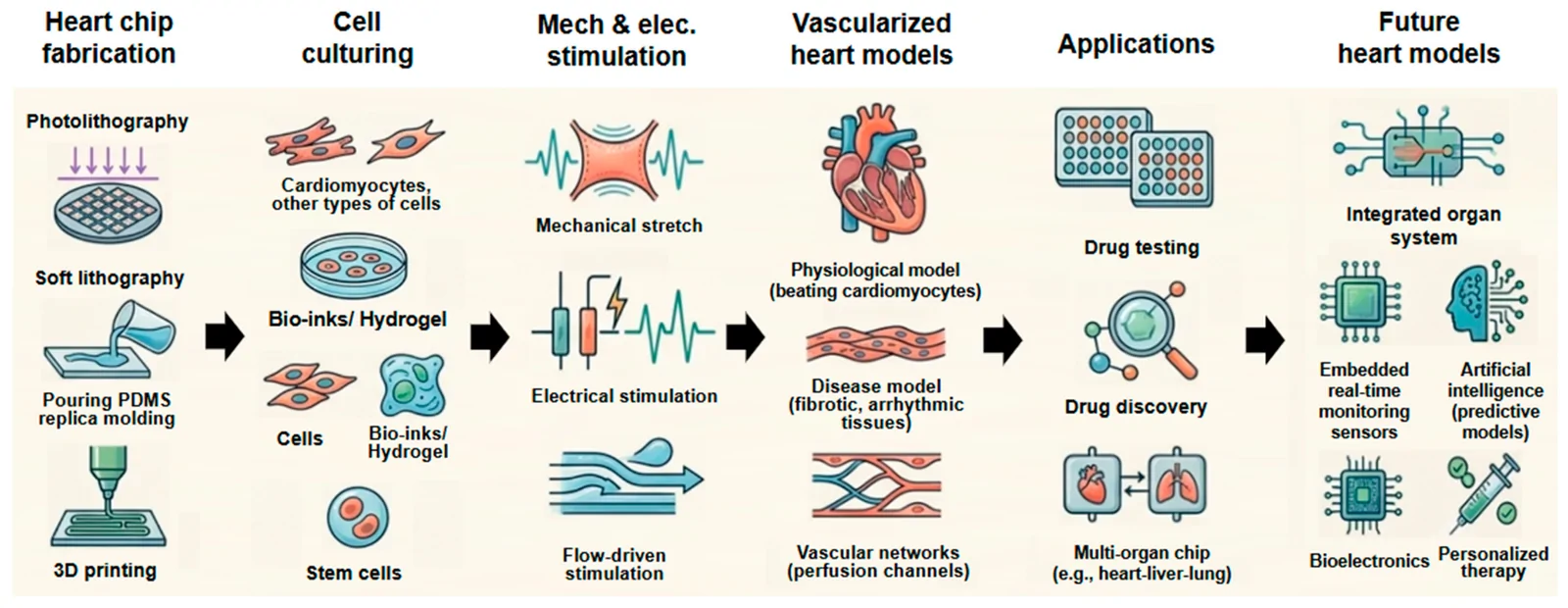
Roadmap for heart-on-a-chip development. Heart-on-a-chip platforms are developed by combining initial microfabrication and cell seeding with electromechanical conditioning to yield physiological or diseased, vascularized cardiac models. Ultimately, these advanced heart models will evolve into next-generation, integrated multi-organ systems equipped with real-time biosensors and AI-driven personalized therapeutics.

Because of the limitations of human and animal experimentation, in vitro cardiac models designed in laboratories have been gaining traction. Such models include 3D engineered heart tissues, cardiac organoids, 2D monolayer cultures, biomaterial scaffolds, and heart-on-a-chip. Figure 1 schematically outlines the development roadmap for a heart-on-a-chip system. A heart-on-a-chip (HoC) is a microphysiological device specifically engineered to mimic the structural, mechanical, and biochemical microenvironment of the native heart [2,3]. By integrating microfluidics with living cellular architectures, these chips allow researchers to study heart disease and model complex cardiac mechanisms with unprecedented accuracy right on a laboratory benchtop.

HoC is a microfluidic device used to culture and observe cardiac cells, aiming to recreate the heart’s physiological, electrical, and mechanical systems [4,5]. HoC allows researchers to control electrical stimulation, contractility, oxygenation, and more, all while allowing for real-time observations [6]. The device can easily be personalized for drug testing, patient cases, and disease modeling due to the high adaptability and variability of cells.

While HoC has many advantages, several critical bottlenecks still exist within current HoC designs. The immaturity of cardiomyocytes and other cardiac cells remains a prevalent issue across most studies conducted, as lab-grown cells still lag behind adult phenotypes [2,5]. Additionally, many devices lack the integration of multiple cardiac cell types and immune cells, and sometimes utilize non-human cardiac cells, which limits their translational accuracy to human patients. Furthermore, there is a lack of functional, perfusable vascular networks in HoC models. Finally, existing HoC systems heavily rely on pillar deflection to measure contraction forces, a method that unfortunately only captures global force at the interface rather than localized tissue mechanics [2,7]. We reviewed the recently developed heart-on-a-chip. In this review, we aim to provide an overview of the currently developed heart models and their applications, challenges, and future perspectives. This paper is organized to support the HoC in different ways, by serving as both a framework for those who are unaware of the HoC in addition to those who specialize in it.

## 2. Fabrication and Stimulation of the Heart Chips

### 2.1. Device Fabrication

Replicating the cardiac microenvironment requires researchers to select suitable biomaterials capable of supporting cell culture, reproducing in vivo conditions, and maintaining the structural integrity of the device (Table 1). Alongside material selection, employing appropriate manufacturing methods is critical to successfully replicating the microenvironment, microfluidic channels, and complex geometric structures necessary for a precise HoC. Several microfabrication techniques are commonly utilized, including photolithography, soft lithography, 3D printing, 3D bioprinting, and laser cutting [8,9]. Among these, photolithography and soft lithography remain the most widely adopted methods [10]. Veldhuizenet et al., among many, utilized SU-8 photolithography to create silicon masters, soft lithography with oxygen plasma bonding to fabricate the chips, and collagen/matrigel-based hydrogel encapsulation for in-chip formation of 3D cardiac tissue and maturation of functional three-dimensional cardiac tissue capable of synchronized contraction [11].

However, photolithography and soft lithography do not have to be used solely on their own. They can often provide a structural foundation into which other components can be incorporated. Researchers are then able to more accurately recreate the in vivo microenvironment and inherent heterogeneity. For example, Wang et al. fabricated their device using soft lithography and a polydimethylsiloxane (PDMS) stamping method, using the muscular thin film heart-on-a-chip assay to model Barth syndrome cardiomyopathy. Thin elastomeric PDMS substrates were micropatterned with fibronectin lines using microcontact printing to guide induced pluripotent stem cell-derived cardiomyocyte (iPSC-CM) alignment into anisotropic laminar myocardium. iPSC-CMs from Barth syndrome patient-derived cells were seeded onto these fibronectin-micropatterned PDMS coverslips. iPSC-CMs self-organized into laminar, anisotropic myocardium recapitulating the architecture of the native ventricular wall and were cultured for 5 days [21].

Some devices were fabricated in combination with 3D printing and soft lithography [59,60,61]. Lind et al. used six functional materials with tunable mechanical and electrical properties to print an integrated device in a single continuous automated procedure [24]. This approach eliminated the need for multi-step lithographic processes and enabled rapid customization of device geometry, enabling long-term contractile monitoring inside an incubator environment via electronic resistance readout. Similarly, Ji et al. fabricated a cardiac fibrosis-on-a-chip with three PDMS layers through 3D wax printing and PDMS casting [62].

Unlike typical 3D printing, 3D bioprinting works with bio-inks, a combination of living cells and hydrogel, instead of printing plastic [8]. Wu et al. created a fabrication method to easily make HoC platforms through 3D bioprinting and hot embossing. A blank polystyrene sheet and carbon electrodes were placed closely on both sides of the microwell pattern on the PDMS mold affixed to the silicon wafer. Then, the polystyrene melted around the carbon electrodes, permanently bonding them together. On this base, thermoplastic elastomer/quantum dot (TPE/QD) nanowires were 3D bioprinted. This process eliminated the manual insertion of wires while simplifying fabrication and improving electrical integration [39]. In another study, Liu et al. also developed a heart-on-a-chip platform using direct 3D bioprinting. Cardiomyocytes were mixed with gelatin methacrylate (GelMA) hydrogel and printed using Microscale Continuous Optical Printing (μCOP). The printed cardiac microtissues were supported by a cantilever-based scaffold that promoted cardiomyocyte alignment and tissue formation [26].

Laser cutting also works alongside PDMS replica molding in developing a HoC, becoming increasingly popular by enabling rapid phototyping while reducing manufacturing complexity [50,56]. For example, Ahn et al. fabricated a mussel-inspired HoC device, beginning with a PDMS block on top of a gelatin-MTG mixture. After curing, the PDMS was carefully lifted off the gelatin surface. The cured gelatin substrates were then coated with polycaprolactone diacrylate (PCL/DA) fibers. Each HoC was then laser-cut, sterilized with UV light, and finalized by being fiber-coated with polymer solution-based nanofibers [38]. Alternatively, Lee et al. designed a PMMA mask through laser cutting and developed a PDMS mold. A negative silicon mold was made using standard lithography to generate the PDMS micro-well platform [63].

Lastly, some researchers used CNC milling and hot embossing methods to create the device actuation or template for molding [39,64]. Tadevosyan et al. built a heart-on-a-chip using CNC-machined COP plastic and PTFE spacers with collagen/Matrigel hydrogel cell tissue. A perfused microfluidic pumping system along with a porous membrane sandwich was also utilized [16].

Each microfabrication technique used in HoC systems fills a distinct role in replicating artificial heart tissue by balancing resolution, throughput, and material constraints. Photolithography excels in sub-micron precision for silicon or glass master patterns, but it is constrained by extreme cleanroom costs and poor direct biocompatibility. Soft lithography offers an accessible, highly biocompatible alternative for rapid benchtop elastomeric casting such as PDMS devices, though its soft polymer matrices can absorb hydrophobic molecules. 3D bioprinting stands alone in its ability to deposit living cells and bio-inks directly into 3D architectures [8,39], but it trades away structural resolution and manufacturing speed. Laser ablation provides rapid, maskless direct-write patterning across diverse materials without chemical additives, yet it risks localized thermal damage and surface roughness [63]. CNC milling enables rapid, low-cost prototyping directly in rigid, medical-grade thermoplastics, though its feature sizes are fundamentally limited by physical cutting-tool dimensions [16]. Hot embossing requires substantial upfront tooling costs for precision stamp creation, but it offers unparalleled throughput and low per-unit costs for mass-producing disposable polymeric microfluidics.

Collectively, studies across a wide variety of strategies demonstrate that integrating photolithography and soft lithography alongside other components serves as a foundational approach in many HoC designs, significantly increasing device complexity and functionality while better mimicking the in vivo microenvironment [36].

### 2.2. Mechanical Stimulation

Heart tissues are dynamic, heterogeneous, and highly complex structures that exhibit microfluidic environments, constant mechanical motion, and electrical stimulation. Integrating mechanical and/or electrical stimulation systems into the device is crucial, as their synergy significantly enhances cellular maturation and function. This stimulation recreates a dynamic, in vivo-like microenvironment within the chip.

**Figure 2:**
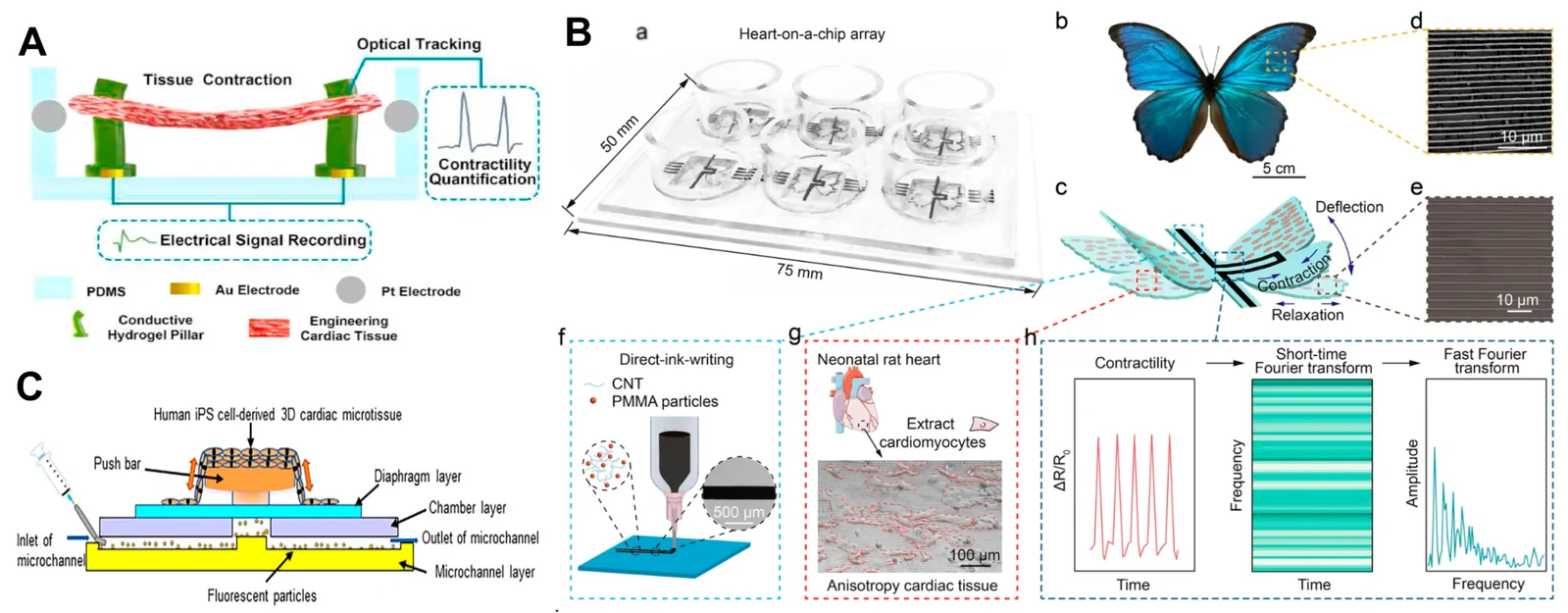
Mechanical stimulation of the heart chip. (**A**) Schematic of the heart chip including PDMS culture chamber, Pt rods, and glass base integrated with Au and conductive hydrogel pillar electrodes [68]. (**B**) The heart-on-a-chip platform inspired by butterfly wings incorporates microgroove structures and an integrated strain sensor that increases strain sensitivity and bandwidth while reducing damping, thereby enabling high-fidelity detection of cardiomyocyte contractility [69]. (**a**) Physical map of a HOC array. (**b**) Optical image of a butterfly. (**c**) Schematic showing the working principle of a strain sensor. (**d**) Scanning electron microscopy image of the wing surface of butterfly. (**e**) The fabricated micro-grooves. (**f**) Schematic showing the direct ink writing of CNT-PMMA as the sensing element. (**g**) Cardiomyocytes were cultured on micro-grooves. (**h**) Relative change in resistance caused by deflection of butterfly-like wing substrate and Fourier transform analysis. (**C**) Schematic of the microfluidic heart chip to stimulate the human iPSC-derived 3D cardiac microtissues by pushing up and down a bar through a microfluidic channel [2]. Images reproduced with permission, from [2,56,68].

To achieve mechanical stimulation, stretching or compressive mechanical mechanisms are typically established on the platform through various ways, including structural scaffolds and pneumatic or mechanical actuation (Table 1, Figure 2) [15,27,62,65,66]. As a common approach, Abulaiti et al. utilized a PDMS diaphragm and push bar assembly to apply mechanical compression to cardiac microtissues [2]. Pneumatic actuation of the diaphragm deflected the push bar to compress tissues, mimicking cardiac mechanical loading. Particle displacement within microchannels was used to quantify stroke volume, pressure, and contractile force non-invasively. Similarly, Kong et al. successfully performed pneumatic mechanical stimulation on a microphysiological system (MPS). The microdevice was tri-layered, with an N2 pressure chamber on the bottom, a medium chamber in the middle where the hydrogels (e.g., a methacrylate GelMA disk) would be integrated, and a glass cover on top. The MPS was put under biaxial, cyclical strain for a week using gas pressure, with the gradient strain being denoted as 0, 5, 10, 15, or 20%. Actuation pressure was measured between 14 and 35 kPa, and cyclic frequency was between 1 and 2 Hz. Naturally physiological strains (5–10%) could maintain or weaken myofibroblast activation, while abnormal pathological strains (15–20%) would allow the phenotype transition towards myofibroblast, thus demonstrating the significance of mechanical stimulation in cardiac fibroblast phenotypic remodeling [67].

To measure the amount of mechanical stretching and contraction, Lind et al. integrated a piezoresistive TPU strain sensor that measures contractile deflection via an electronic resistance readout, enabling long-term monitoring inside an incubator [24]. To further improve electrical monitoring, Visone et al. developed a human beating heart-on-chip platform using micro-electrode channel guide (µECG) technology to enable direct electrical recording from 3D cardiac microtissues. The h-iPSC cardiomyocytes and human fibroblasts, guided by PDMS microchannels and mechanically stimulated with 10% uniaxial strain at 1 Hz, formed synchronized beating tissues after 7 days. The cardiac microtissues generated measurable field potentials with a beating period of 1.7 ± 0.45 s and spike amplitude of 590 ± 440 µV. Drug testing showed that sotalol prolonged corrected field potential duration by up to about 80%, while verapamil reduced it by up to about 40%. This demonstrates that direct electrical recording in mechanically trained 3D heart-on-chip models can improve cardiotoxicity testing and detection of drug-induced electrophysiological changes [12].

Some researchers focused on building tissue architecture so that its structural scaffold or pattern may induce cells to create contractile and stretching motion [15,43,45,47,51,63,70,71,72]. Specifically, Sheehy et al. did not employ electrical or mechanical stimulation, but instead focused on tissue architecture [45]. The surface of a PDMS muscular thin film was patterned with parallel lines of fibronectin. Then, neonatal rat cardiomyocytes were seeded onto the entire substrate, but the cardiomyocytes preferentially adhered to fibronectin lines. This created an anisotropic tissue organization. In vitro, an iso-line-engineered myocardium displayed a high degree of uniaxial, parallel z-line sarcomeric organization similar to that of an adult rat ventricular myocardium. In addition, the highly aligned engineered tissues exhibited greater contractile force, comparable to adult ventricular muscle strips. In the study by Kołodziejek et al., they created a device where cardiomyocytes were cultured on a nanofibrous scaffold that provided cyclic mechanical stimulation (0.5 Hz, 1 h/day for 5 days) by deforming embedded magnetic nanoparticles to deliver mechanical cues to the cells [51].

Morimoto et al. used a passive mechanical loading method to make fiber-shaped 3D cardiac constructs using hiPS-CMs [13]. They used a PDMS stamp to shape a hydrogel made of Matrigel and type I collagen with cells inside it into narrow and wide fiber shapes. This created a constant passive tension along the fiber. The narrow fibers (0.5 × 0.5 × 5 mm) had better cell alignment than the wide ones or the unconfined clumps. After 14 days, the narrow fibers showed higher expression of cardiac markers like α-actinin and cTnT. They also had stable spontaneous beating at around 0.74 Hz. The contractile frequencies of the hiPS-CM fibers were synchronized with the frequencies of electrical stimulations to improve the overall contractile output.

Likewise, numerous heart-on-a-chip platforms have adopted various mechanical stimulation mechanisms, including the use of structural scaffolds and actuators to apply physical forces to the cardiac tissues by inducing controlled mechanical deformation with a specific pulse, cycle, and duration. The resulting data demonstrate a significant contribution to the enhancement of cardiac cell beating and maturation driven by these contractile and stretching cues.

### 2.3. Electrical Stimulation

**Figure 3:**
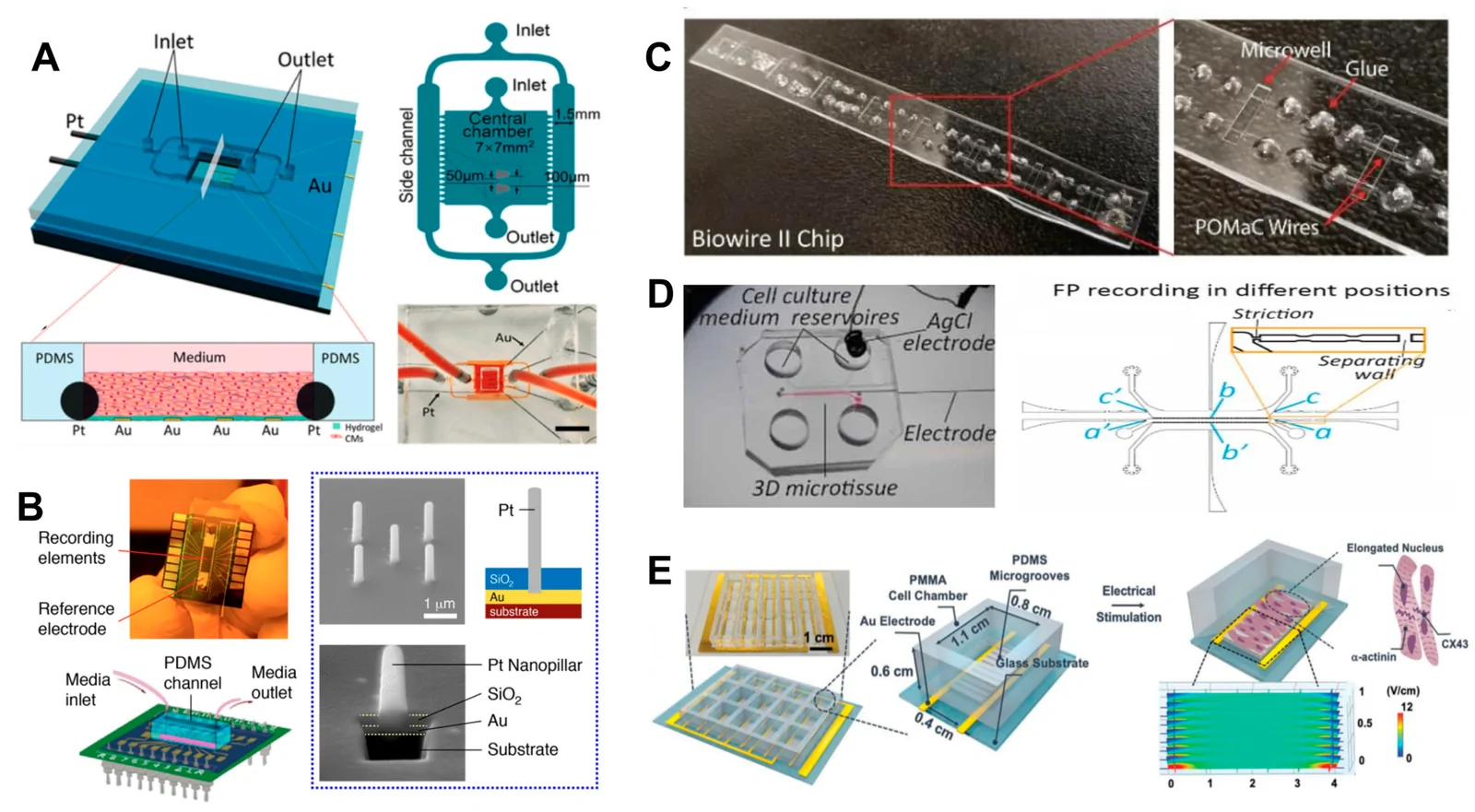
Electrical stimulation of the heart chip. (**A**) The heart chip demonstrates the design of the PDMS microfluidic device [55]. Scale bar: 1 cm. (**B**) Portrays the fully assembled chip with a PDMS channel, recording elements, and a reference electrode [40]. (**C**) The schematic illustrates the Biowire II heart-on-a-chip platform [50]. (**D**) The device uses micro-electrocardiogram channels to guide electrodes into designated regions of 3D cardiac microtissues, facilitating targeted field potential signal recording [12]. (**E**) The cardiac tissue-on-a-chip is fabricated using microgrooves and electrical stimulation, highlighting how these features synergistically enhance cardiac tissue development and functional maturation [35]. Images reproduced with permission, from [12,35,40,50,55].

Electrical stimulation guides the immature cells to compact, remodel, and align, ultimately encouraging mature function and synchronized beating that mimics adult heart tissue. To deliver electrical stimulation or detect electrical signals, electrodes are placed on the chips to provide the input signals and detect the responding signals from the tissue (Figure 3) [17,18,55,73]. While metal electrodes can be simply inserted into the device, creating patterned configurations like microarrays represents a more advanced integration method [13,42,49]. Recently, researchers have printed electroconductive metal on a device using 3D printers. Operating chip-integrated electrodes effectively requires both optimizing key stimulation parameters such as cycle, pulse amplitude, and duration and measuring the tissue’s subsequent feedback response (Table 1).

A couple of decades ago, researchers frequently utilized metal wire electrodes or the Biowire platform as a gold standard for electromechanical conditioning by providing direct electrical input to the heart tissue. Cofiño-Fabres et al. placed two platinum electrodes in the two inlets of the chip to electrically stimulate micro-engineered heart tissues [49]. A custom-made pacing device began operating at 1.5 Hz (10 ms biphasic pulses, 3 V/cm) and increased the frequency by 0.5 Hz every 20 s. This resulted in a HoC model with polarized formation of 3D heterotypic human cardiac tissues. In another study, Morimoto et al. tested electrical stimulation on their hiPS-CM fibers. Gold electrodes were used to apply electrical pulses to the fiber constructs [13]. The pulse conditions were electric field: 1.2 V mm^−1^, duration: 20 ms. The fibers responded by synchronizing their beating with the applied frequency, which is something that real heart tissue also does. They also found that electrically stimulated fibers had a higher peak-to-peak contractile stress than fibers that were just beating spontaneously. The Biowire platform utilizes a sterile silk suture to form a structural scaffold and provide mechanical boundaries [52,55]. Wagner et al. created the Biowire platform using soft lithography to create molds for plate microwells, and hot embossing of polystyrene with embedded carbon electrodes to transfer the mold pattern [53].

In recent research, Biowire II serves as an upgraded platform of Biowire that incorporates two flexible, elastic polymer wires instead of a silk suture [14,50,52]. As the tissue contracts, it pulls and bends these wires. By tracking the pixel displacement or deflection of the wires under a microscope, the system quantifies twitch force and contractile output in real time without damaging the tissue. Consequently, these systems provide a powerful tool for modeling chamber-specific (e.g., atrial or ventricular) tissues, conducting preclinical drug screenings, and developing high-fidelity disease models.

For example, Feric et al. used a Biowire II HoC platform to engineer cardiac tissue from hiPSC-CMs and cardiac fibroblasts, using electrical stimulation [74]. The engineered cardiac tissue (ECT) containing the cardiomyocytes and cardiac fibroblasts was strung between PoMAC poly(octamethylene maleate (anhydride) citrate) wires. Custom-made parallel carbon electrodes were used to electrically stimulate the cells for a duration of 2 ms at 1 Hz, increasing by an increment of 0.1 Hz daily over the course of 6 weeks to a final maximum of 6 Hz. The ECTs displayed human adult-like contractile abilities, showing a positive force-frequency relationship and prominent post-rest potentiation after being electrically stimulated for 6 weeks. Bannerman et al. also developed HoC constructed using the Biowire II platform to incorporate epicardial cells alongside cardiomyocytes and fibroblasts [14]. It used electrical pacing only as a diagnostic tool to measure the excitation threshold and maximum capture rate, and mechanical properties were tracked via wire deflection. Over time, the maximum capture rate declined for both because of the absence of electrical conditioning, which demonstrated that mechanical and electrical maturation can decouple, so sustained electrical capture may specifically require continuous electrical stimulation rather than just tissue maturation.

One way to create a specific desired electrode pattern is to use microfabrication technology such as a microelectrode array (MEA) [18]. In the study by Liu et al., he integrated the HoC with a MEA stimulation system and intracellular probes consisting of platinum nanopillar arrays [40]. The main purpose of MEA and intracellular probes was not chronic maturation of cardiac cells but rather obtaining a continuous readout of cardiac signaling. Through this, the engineered tissues followed controlled pacing, and the authors were able to measure conduction velocity for normoxia and acute hypoxia. Choi et al. also desired to precisely model human cardiovascular function for accurate cardiotoxicity detection in drug testing [75]. In order to achieve this, an hiPSC-CM-based HoC device was created based on a custom-made multi-electrode array (cMEA). The inner structure of the HoC consisted of perimysium-like collagen layer channels containing myocardial fibers, with electron probes placed in the chamber. Isoproterenol, which increases heart rate, and nifedipine, which decreases heart rate, were used as the drugs responsible for controlling the heart rate during the cardiotoxicity testing. Additionally, alterations in electrocardiogram waveforms, such as those in T-wave amplitude and QT interval, were recorded. T-wave amplitude slightly increased when exposed to isoproterenol and decreased significantly when exposed to nifedipine. The QT interval decreased under both conditions.

In recent years, Esmaeili et al. fabricated an electroconductive HoC environment to investigate human cardiovascular response to gold nanomaterials [43]. Firstly, golden nanorods (GNRs) were incorporated into hydrogel scaffolds in the HoC device for their electroconductive properties. Essentially, there was no external electrical stimulation, with the GNRs being the sole source of electroconductivity. The hydrogel scaffolds were frozen in liquid nitrogen, freeze-dried, and coated in gold. In order to evaluate the impedance, the resistance to an alternating current (AC), an electrochemical impedance spectroscopy (EIS) potentiostat equipped with 2 electrodes (probes) was utilized. From 1 Hz to 100 kHz, a voltage of 10 mV was applied to the cells, and it was found that the hydrogel scaffolds containing GNRs experienced less impedance, thus meaning that electrical signals can move through the system more efficiently. Additionally, GNR integration improved hiPSC-CM maturation and synchronization. In the research by Sun et al., they developed a conductive and anisotropic structural color hydrogel [9,73]. The hydrogel consisted of two sides. One side was made of GelMA/super-aligned carbon nanotube sheets (SACNTs) with electroconductive properties, and the other was a colloidal array-locked hydrogel layer, possessing structural color properties. Additionally, bending of the cardiomyocyte cells induced the color-changing, anisotropic effect. In terms of the electroconductive properties, silica nanoparticles were integrated into the hydrogel solution to make the hydrogel conductive, allow the synchronous beating of cardiomyocytes, and enhance cell alignment. The spontaneous contraction of the cardiomyocyte cells acts as an indirect form of mechanical stimulation.

Another way to create electrical patterns is to print electroconductive inks, including liquid metal, on the device using 3D printers. Lind et al. developed a cardiac microphysiological device using multi-material 3D-printing with a soft PDMS, TPU ink, and silver-polyamide (Ag:PA) ink [24].

In summary, the platforms for electrical stimulation have driven significant advancements by delivering precise electrical input and measuring functional output, seamlessly linking tissue motion with physiological performance in the development of biomimetic cardiac tissues. Consequently, applying electrical stimulation through these devices has greatly enhanced heart (patho)physiological modeling by successfully inducing physiological cardiomyocyte beating and promoting the expression of cardiac tissue markers.

### 2.4. Both Mechanical and Electrical Stimulation

Integrating both mechanical and electrical stimulation is more complex and requires more advanced fabrication and handling technologies (Table 1) [25]. Zhang et al. used both electrical and mechanical stimulation in a HoC [68]. Gold electrodes with two hydrogel pillar electrodes were fitted inside the HoC platform. The conductive hydrogel was fabricated using a gelatin-powder mixture, and the pillar electrodes were fabricated by PMMA and PDMS molding, with the pillar adhering to the surface. For electrical stimulation, a cardiac stimulator was hooked up to the cardiac tissue with platinum wires. The stimulation on the tissue began once the cells were cultivated for 1 day, being stimulated at 3–4 V for 2 ms at 2 Hz, with the frequency being increased by 1 Hz daily until 6 Hz was reached. For mechanical stimulation, the tissue was stretched uniaxially from natural cardiomyocyte contraction once tautly attached to the hydrogel pillar. Similarly, Wu et al. developed a heart-on-a-chip platform that used conductive hydrogel pillars to continuously monitor both the contractile force and electrical activity of three-dimensional cardiac tissues [59].

Manjua et al. also developed an organ-on-a-chip with tri-stimulation (electrical, mechanical, and magnetic) accommodating iPSC-CMs and human umbilical vein endothelial cells (HUVECs) [76]. Electromagnetic scaffolds were fabricated by coating electroconductive poly-(3,4-ethylenedioxythiophene) polystyrenesulfonate (PEDOT:PSS) fibers, consisting of a polycaprolactone (PCL) shell and a hydrogel center containing electroconductive iron oxide nanoparticles (MNPs). Mechanical stimulation was indirect, with the hydrogel’s shape being altered through magnetism, thus modeling cellular response to mechanical interactions. In terms of electrical stimulation, an AC/DC power source with four platinum electrodes was connected to the PDMS chip, and a voltage between 0 and 10 V at 3 Hz was applied for a duration of 24 h. Then a neodymium magnet was placed underneath the device, causing electromagnetic stimulation to the cells. Such electromagnetic stimulation was found to restore cardiac contraction, which may potentially open opportunities for research on cell remodeling or repair.

### 2.5. Sensing and Detection Modalities in HoCs

HoC platforms need reliable detection methods to evaluate functional responses of built cardiac tissues to mechanical and electrical stimulation. In situ detection methods allow real-time monitoring of cardiac tissues without removing them from the device. Mechanical responses can be measured through tissue displacement, deformation, strain, or contractile force, while piezoresistive sensors can convert tissue deformation into electrical signals for monitoring [2,24]. Similarly, the Biowire II platform measures contractile output by maintaining and tracking the displacement of flexible polymer wires during tissue contraction [14,50,52]. Electrical activity can be monitored using MEAs and µECG systems. These systems can measure parameters like field potentials and conduction velocity [12,40]. Other approaches, such as electrochemical impedance spectroscopy (EIS), can assess the electrical properties of engineered cardiac scaffolds [43].

As offline testing methods, microscopy and immunostaining can analyze cardiomyocyte alignment, sarcomere organization, tissue morphology, and cardiac protein expression, while gene and protein expression analyses can give more information about tissue maturation and remodeling [13,45]. Combining these offline approaches with in situ allows functional responses to be correlated with underlying cellular changes. Overall, the application of real-time detection and offline characterization provides a better comprehensive assessment of cardiac tissue responses and strengthens the application of HoC platforms in disease modeling, drug screening, and cardiac tissue maturation.

In summary, heart-on-a-chip systems rely on cellular stimulation because it promotes cell growth, maturation, and overall tissue health, making the engineered tissue behave more like native heart muscle. The implementation of mechanical stretching systems, electrical stimulation platforms, or a combination of both acts as a key driver in enhancing the replication of cardiac functionality and promoting stem cell maturation.

## 3. Current Heart Models and Applications

**Figure 4:**
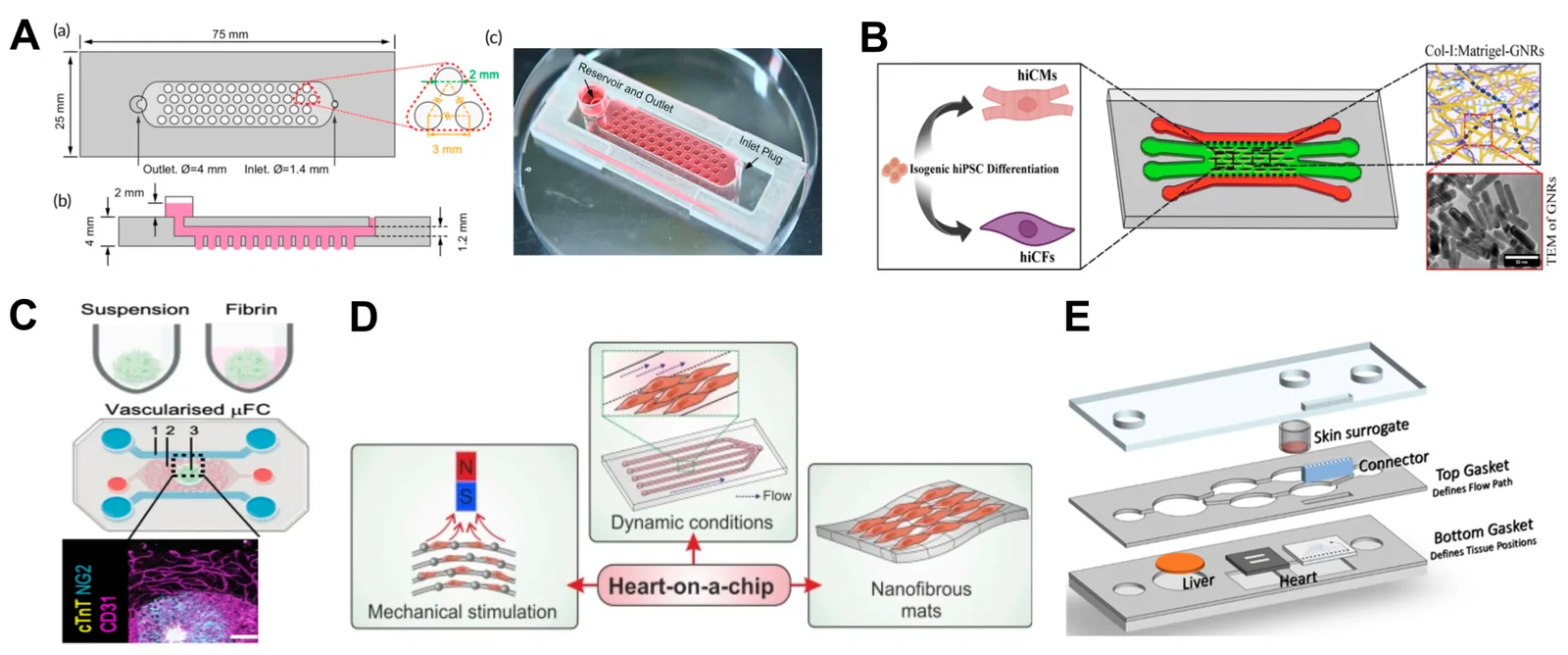
Application of the heart chip. (**A**). The hanging-heart chip enables parallel formation of cardiac spheroids to generate functional, beating 3D heart tissue models for drug testing [27]. (**a**) Top and (**b**) side views of the channel structure. (**c**) Photograph of the device placed within a 10 cm dish. (**B**). The schematic illustrates the heart model, in which isogenic hiPSC-CMs and hiPSC-CFs are seeded on electroconductive hydrogel scaffolds [43]. Scale bar is 50 nm. (**C**). Schematic of the vascularized microfluidic heart-on-a-chip shows medium channels, the vascularized gas chamber, and the central cell culture well [41]. In the device schematic: 1 is the medium channels, 2 is the vascularized gel chamber, and 3 is the central cell culture well for the vascularized spheroid. Scale bar is 100 µm. (**D**). The heart chip combines stimulation, fluid flow, and nanofibrous scaffolds to mimic cardiac tissue [51]. (**E**). The schematics of the heart–liver body-on-a-chip system with the skin surrogate [42]. Images reproduced with permission, from [27,41,42,43,51].

Early cardiac chips began with simple architectures and straightforward handling methods. Specifically, researchers historically attempted to culture non-stem cell lines, such as adult primary heart cells, in either mono-culture or co-culture configurations. These early platforms utilized standard two-dimensional monolayers rather than 3D spheroids and were limited to short-term cultures of fewer than seven days. Over time, these platforms have evolved into much more sophisticated devices. Modern systems frequently support complex triple co-cultures, stem-cell-derived cardiomyocyte integration, and long-term 3D spheroid cultivation (Table 1). For instance, traditional dual-layer channel designs have been upgraded to multi-layered structures capable of housing three distinct cell types simultaneously. Furthermore, these contemporary platforms are routinely integrated with active mechanical and electrical stimulation systems to replicate the physical contractile, stretching, and pumping functions of native heart tissue (Figure 4). As a result, these advanced devices are successfully applied to complex heart disease modeling (e.g., ischemia, fibrosis), drug screening, targeted drug delivery, physiological cardiac modeling, and multi-organ-on-a-chip human body simulations (Table 1) [26,77].

### 3.1. Heart Physiology Modeling

Heart tissues are dynamic, heterogeneous, and highly complex structures that exhibit microfluidic environments, constant mechanical motion, and electrical stimulation. In order to improve the physiological accuracy of Heart-on-a-Chip (HoC) systems, it is essential to closely mimic this in vivo microenvironment and its inherent heterogeneity [27,32,38,78].

#### 3.1.1. Dynamic Co-Culture of Cardiac Tissues and Stem Cell-Derived HoC Platforms

Kobuszewska et al. investigated how static versus dynamic conditions affect the structure, growth, and arrangement of rat cardiomyocytes by using three microsystems [72]. The microsystem consisted of two layers: a glass slide on the bottom and a PDMS plate on top, made using photolithography and replica molding techniques. Culture medium was fed into the system through a peristaltic pump, and then discarded into a waste reservoir at the opposite end. Perfusion conditions greatly increased cell numbers, with the cell population almost doubling. Building on Kobuszewska’s approach, some researchers created multilayer chips to culture multiple types of cells [49,79,80,81]. Tadevosyan et al. fabricated a HoC that contained three different cell types, including induced pluripotent stem cell (iPSC)-derived cardiomyocytes, cardiac fibroblasts, and endothelial cells, and consisted of an upper and lower plate, a spacer, and a disposable cell culture disc [16]. In terms of layout, the cell culture membrane was sandwiched between the stainless-steel lower and upper plates. An endothelial cell layer that creates a vascular line protected against doxorubicin toxicity and replicated the Isoproterenol (ISO) response. By including an endothelial barrier, it also improved cell interactions and more accurately replicated the in vivo environment, which showed that this device is a valid tool to better detect cardiotoxicity in drug testing. In addition to improving functional assessment, HoC platforms have also been designed to increase reproducibility of cardiac tissue production for drug screening. Lai et al. developed a heart chip that uses a microfluidic hanging-drop system, which enables high-throughput generation of contractile embryonic stem cell-derived cardiac spheroids [27]. The hanging-heart chip generated 50 embryoid bodies, with 90% of them becoming beating cardiac spheroids within 15 days and reaching a beating rate of around 60 BPM. Likewise, a hanging-heart chip is capable of improving cardiac tissue modeling and high-throughput drug testing by producing many consistent beating heart spheroids.

Also, hiPSC-HoC platforms serve as powerful biomimetic tools for modeling cardiac developmental biology by recreating key physical and biochemical microenvironments in vitro [16,28]. During early cardiogenesis, microfluidic channels precisely control morphogen gradients such as the Wnt signaling pathway and deliver localized fluid shear stress, guiding stem cell differentiation into spatially organized mesodermal progenitors and cardiac lineages [70,82]. As the tissue develops, the HoC platform applies cyclic mechanical stretching and electrical pacing alongside continuous nutrient perfusion, which actively drives structural maturation by promoting cellular alignment, sarcomere elongation, myofibrillar density, and electrophysiological development [22].

#### 3.1.2. Vascularized Cardiac Tissues

A vascularized cardiac tissue-on-a-chip contains the microfluidic central chamber with cardiac cells and a surrounding system of vascular networks [41]. Seeding cardiac cells and endothelial cells at the same time integrates a vascular system, and HUVECs are sufficient to form a continuous vascular area. The perfusable vascular network better mimics native myocardial tissue, microfluidic phenomena, and mass-transport function.

For example, Mozneb et al. developed a multi-lineage heart-on-a-chip platform that has human iPSC-derived cardiomyocytes and endothelial cells in separate microfluidic channels [28]. The hiPSC-derived cardiomyocytes were placed in the top myocardial channel, while the hiPSC-derived endothelial cells were placed in the bottom vascular channel with active fluid flow and rhythmic biomechanical stretch. This allowed the cardiovascular cells to become more mature, with cardiomyocytes showing improved sarcomere alignment and endothelial cells aligning with the direction of flow. The heart-chips used 30 μL/hour fluid flow, 0.4 Hz rhythmic stretch, and 10% chip deformation, while maintaining beating around 60 BPM for multiple weeks and showing sorafenib-induced cardiotoxicity after 4 days of treatment. Overall, combining multiple human cardiovascular cell types with microfluidic flow and mechanical stimulation is capable of improving heart-on-a-chip models.

#### 3.1.3. Cardiac Electrophysiology

Further improving tissue architecture and maturation, HoC platforms have also been developed to monitor cardiac electrophysiology and improve the detection of drug-induced arrhythmias [32,83]. Marsano et al. developed the I-Wire Heart-on-a-Chip platform, a three-dimensional engineered cardiac tissue model designed for pharmacological studies [70]. The device cultured cardiac cells within a 3D construct suspended between flexible wires, allowing researchers to apply controlled electrical stimulation and measure tissue contractions. The cardiac tissues responded to electrical pacing with synchronized beating and displayed functional properties similar to native heart tissue [83]. Visone et al. developed a 3D beating heart-on-a-chip platform to predict changes in the QT interval and assess the pro-arrhythmic effects of pharmaceutical compounds [84]. The device contained engineered human cardiac tissues that generated spontaneous and synchronized contractions while allowing continuous monitoring of electrical activity. The continuous electrical activity monitoring allowed real-time assessment of the drug-induced changes in cardiac electrical behavior without disrupting the created tissue. The researchers tested several drugs known to affect cardiac electrophysiology and observed changes in beating patterns, action potential duration, and QT-related parameters. The platform successfully identified compounds that increased the risk of arrhythmias, producing results that closely matched known human cardiac responses [85,86]. Similarly, Zhang et al. developed a high-throughput heart-on-a-chip platform to assess the cardiac safety of pharmaceutical compounds [87]. The device used engineered human cardiac tissues and integrated sensors to monitor changes in beating behavior and electrical activity after drug exposure. By testing multiple compounds simultaneously, the researchers were able to identify drugs that caused cardiotoxic effects, including irregular beating patterns and altered electrophysiological responses.

### 3.2. Heart Disease Modeling

There have been many attempts to apply HoC to the study of heart disease, and many efforts to create heart pathology models [36,40]. Gaballah et al. created a cardiac ischemia-on-a-chip model by exposing hiPSC-CMs to hypoxia and glucose deprivation [88]. The ischemia impaired the contractile performance of cardiomyocytes and induced arrhythmias, typical of symptoms of ischemia in the human body. The chip was then treated with Levosimendan, which reduced ischemia-induced arrhythmias. This platform displayed promising results in the screening of anti-arrhythmic drugs. In addition to a vascularization strategy, Kim et al. developed the perfusable HoC to model myocardial ischemia and evaluate targeted nanomedicine delivery [17]. The device utilized co-cultured hiPSC-derived cardiomyocytes, cardiac fibroblasts, and endothelial cells in a fibrin hydrogel, alongside self-assembled microvascular networks that were in direct contact with the engineered myocardial region. To stimulate ischemia, a vasoconstrictor cocktail of angiotensin II and phenylephrine was continuously infused through the side channels of the device over 7 days. This treatment caused the cardiomyocytes to develop arrhythmic calcium transients and reduced contractile consistency, while the microvascular network showed vessel shrinkage, reduced connectivity, and increased permeability, thus mimicking ischemic heart disease. Schmidt et al. also developed a novel immuno-heart-on-a-chip model to study how polarized macrophages affect cardiac tissue under hypoxic conditions [89]. The device incorporated cardiac cells and different macrophage populations within a microfluidic platform, allowing researchers to examine interactions between immune cells and heart tissue. Under hypoxia, the presence of macrophages significantly influenced tissue structure, contractility, and cellular responses. Pro-inflammatory macrophages were associated with reduced contractile function and greater tissue damage, while anti-inflammatory macrophages helped preserve cardiac structure and function.

In another disease modeling study [90], Ji et al. designed a novel cardiac fibrosis-on-a-chip that successfully co-patterned cardiac cells and immune cells [62]. The authors utilized stepped tissue chambers to separate iCMs from iCFs and a valve actuation system for targeted delivery of immune cells and TGF-ß cytokines. This allowed the replication of the heterogeneous tissue architecture of post-myocardial infarction with healthy, border, and scar regions. Wang et al. created a heart-on-a-chip to model the mitochondrial cardiomyopathy associated with Barth syndrome [21]. The researchers engineered cardiac tissues from patient-derived iPSCs and cultured them within a heart-on-a-chip platform to evaluate their structural and functional properties. Barth syndrome tissues exhibited weaker contractions, impaired sarcomere organization, and abnormal mitochondrial function compared to healthy controls. Wu et al. developed an angiotensin II-induced heart-on-a-chip model to investigate the effects of SARS-CoV-2 on diseased cardiac tissue and evaluate potential treatments [91]. The platform contained human cardiac cells exposed to angiotensin II to mimic cardiac injury and disease conditions before viral infection. Following SARS-CoV-2 exposure, the tissues exhibited increased inflammation, cellular damage, and impaired cardiac function, reflecting key features of COVID-19-related heart complications. The researchers also screened extracellular vesicles as a potential therapy and found that they reduced inflammatory responses and improved tissue health.

By replicating structural complexity, tissue functionality, and intercellular interactions, these cardiac models serve as robust platforms for investigating underlying disease pathogenesis and cellular mechanisms.

### 3.3. Drug Testing and Screenings

Most heart models have been applied to drug toxicity studies [16,28,63]. Wu et al. fabricated a multiwell HoC platform-printing device for drug testing with long-term electrical stimulation [39]. Compared with the control, a manually assembled Biowire II HoC, the 3D-printed HoC proved to have sufficient sarcomeric alignment of contractile proteins after 5 weeks of culturing and electrical stimulation, proving its legitimacy. In terms of drug testing, nifedipine and lidocaine were introduced to the 3D-printed HoC, inhibiting contractile force and Ca^2+^ transient levels in the tissue and contraction force, respectively. In another study, Visone et al. developed a beating heart-on-a-chip platform using µECG technology to enable direct electrical recording from 3D cardiac microtissues [12]. Drug testing showed that sotalol prolonged corrected field potential duration by up to about 80%, while verapamil reduced it by up to about 40%. This demonstrates that direct electrical recording in mechanically trained 3D heart-on-a-chip models can improve cardiotoxicity testing and detection of drug-induced electrophysiological changes. A microfluidic heart-breast cancer-on-a-chip incorporating human iPSC-derived cardiac tissues and 3D breast cancer tissues was used for drug testing. Doxorubicin (DOX), a chemotherapy drug, was administered on the chip, which decreased tumor growth and cancer cell viability. The chip also revealed cardiotoxic effects of DOX, such as reduced contractile function and beating amplitude of the cardiac tissue. Additionally, electrochemical immuno-aptasensors detected early biomarkers related to cardiac injury from DOX and cancer progression. The results also showed that the nanoparticle formation of DOX reduced cardiotoxicity compared to DOX [63].

### 3.4. Multiple Organ on a Chip

One of the applications of HoC is to develop multiple organ-on-a-chip systems. Pires de Mello et al. developed a microphysiological multi-organ-on-a-chip, involving skin, liver, and heart tissue on the same device [42]. The chip effectively served its purpose of drug testing by mimicking the drug administration, metabolism, and response of a human body. An engineered Strat-M membrane acted as a drug barrier, allowing the controlled exposure of drugs into the liver-heart system. The liver tissue metabolized drug compounds before they reached the cardiac tissue. This chip closely simulated drug administration in the human body, and the researchers could study the cardiotoxicity of the tested drugs with higher accuracy. Lee et al. developed a heart–breast cancer-on-a-chip platform to model interactions between breast cancer and cardiac tissue and to study chemotherapy-induced cardiotoxicity [63]. The microfluidic device connected engineered heart tissue and breast cancer cells, allowing researchers to examine how cancer treatments affect both tissues simultaneously. After exposure to chemotherapy drugs, the cardiac tissue exhibited reduced contractility, altered beating behavior, and signs of cellular damage, while the cancer tissue responded to treatment as expected. The platform enabled real-time monitoring of drug effects and revealed the harmful impact of chemotherapy on heart function.

In summary, heart-on-a-chip platforms have been applied across diverse fields, including physiological tissue modeling, disease modeling, cardiotoxicity screening, and multi-organ-on-a-chip integration. Despite persistent technical limitations and engineering challenges, these devices have steadily evolved through advancements in structural scaffolding, electromechanical stimulation systems, and biomimetic replication fidelity. Moving forward, the demand, clinical relevance, and applications of these platforms are expected to grow continuously.

## 4. Limitations, Challenges, and Future Perspectives

This review outlines recent research on heart-on-a-chip platforms designed to replicate cardiac architecture, functionality, and the microfluidic microenvironment. As fabrication technologies have advanced and novel biomaterials have emerged, both device design and stimulation modalities have significantly progressed. However, notable limitations and challenges remain in engineering biomimetic cardiac tissues. These hurdles include low reproducibility, high manufacturing costs, an imbalance between oversimplified and overly complex fabrication methods, the structural and functional immaturity of stem cells, suboptimal cell density, inadequate electrical or mechanical stimulation, and a general lack of cellular and therapeutic diversity.

### 4.1. Lack of Reproducibility

Reproducibility is crucial in scientific research, especially in novel engineering of heart-on-a-chip. However, lack of reproducibility has been a long-standing limitation of HoC research. Relatively minor changes that come from the culture environment, culture media, human handling, and unpredictable cell behavior could produce major discrepancies between experimental results. Moreover, some fabrication methods are overly simplified or complex. For example, Ji et al. developed a 3D cardiac fibrosis-on-a-chip that incorporated hiPSC-derived cardiomyocytes and cardiofibroblasts at certain ratios [62]. This allowed accurate spatial organization (healthy, border, and scar regions), an immune cell gradient pattern, and cardiac cell crosstalk. However, building a three-layer microfluidic co-culture device added unprecedented complexity, so achieving reproducible patterning may prove challenging.

### 4.2. Immaturity for Stem Cells

Immaturity of stem cells, such as hiPSC-CMs and hiPSC-CFs, has been a significant limitation in developing hearts-on-a-chip [75]. In the HoC study by Tadevosyan et al., the iPSC-derived cardiomyocytes were immature compared with adult cardiomyocytes and lacked certain attributes that mature cardiomyocytes possess. Unfortunately, the duration of culturing required to mature iPSC-derived cardiomyocytes is time-consuming, generally taking at least a month, and currently there are no long-term solutions to this issue [16]. Other researchers encountered a similar problem [20,21,75]. In the study using the HoC with an integrated multi-electrode array (cMEA), ideal hiPSC-CM maturity was not obtained, which lacks certain parameters such as an elongated rod cell shape, directional alignment of myofibrils, and sarcomere formation [75]. Using more mature hiPSC-CMs, achieved through metabolic or electrical conditioning before co-culture, would help cut down on automaticity and make the electrophysiology more representative [20]. To achieve fully matured adult cells, researchers often employ mechanical and electrical stimulations and design 3D culture devices. While complete maturation of stem cells is still a difficult limitation to overcome, adjusting stimulations and device design may mitigate the issue to a certain point.

### 4.3. Lack of Vascularization

By delivering essential oxygen and nutrients to sustain long-term tissue viability, vascularization enhances the physiological relevance and functional accuracy of heart-on-a-chip models, while the integration of endothelial cells further promotes crucial cardiomyocyte crosstalk [65]. Despite these advantages, lack of vascularization stands as a limitation. To grow endothelial cells, which form blood vessels, the needs of cardiomyocytes may be neglected. Even if a vascular system is created, its stability is not guaranteed. Microvessels are prone to rupture when subjected to mechanical or electrical stimulation of the chip. Lu et al. attempted to use a vascularized heart-on-a-chip to model SARS-CoV-2-induced acute myocarditis [47]. However, microvasculature was absent in the parenchymal space, where iPSC-derived cardiac tissue was cultured. In some studies, a limitation was that vasculogenesis depended on the endothelial cells spontaneously self-organizing in response to VEGF [20,65,92]. Although the resulting vessel network was perfusable, it lacked the precise architecture found in real myocardial capillary beds. Some researchers demonstrate long-term culture for up to eight months for modeling polygenic disease, but staying consistent over that long of a timeframe is still a practical challenge. Adding vascular channels and fibroblast co-culture would also help improve how physiologically relevant the model is [23,93,94].

### 4.4. Lack of Incorporating Multiple Cell Types

The human heart is composed of a number of different cell types: cardiomyocytes, fibroblasts, endothelial cells, immune cells, pericytes, and more. However, most HoCs focus on a singular cell type, usually cardiomyocytes that perform the main function of the heart. Unfortunately, the heart-on-a-chip cannot fully replicate heart physiology without incorporating multiple cell types [46,87]. For example, Ji et al. successfully created an immune-integrated cardiac fibrosis-on-a-chip with accurate patterning of scar, border, and healthy regions of the fibrotic heart [62]; however, one limitation came from primarily focusing on macrophages and neglecting other crucial immune cells such as neutrophils and T-cells. This also led to the absence of endothelial interactions, which are crucial in the post-myocardial infarction period. Cells may facilitate the missing collagen degradation in terms of a complex interplay between fibroblasts, immune cells, and the extracellular matrix [46]. Some heart models also report a lack of cell diversity to replicate the tissue physiology. Therefore, the usage of more cell types, such as progenitor cells, endothelial cells, or epicardial cells, can be potentially beneficial to the accuracy of the research [39,95,96].

### 4.5. Poor Functionality and Stimulations

The primary limitation of the physiological heart model is its reliance on cardiomyocytes or cardiomyoblasts, validating whether its mechanical and electrical functions match native human tissue [18,22,23,36,39,48]. Some microdevices are limited by a very low contractile output (e.g., 0.04 mN) from the immature iPSC-CM microtissues, raising concerns over whether its measured force-frequency relationship accurately reflects native adult myocardium [2,23,97,98]. Despite achieving impressive structural and molecular maturity, the platform remains limited by a geometric mismatch between its cylindrical tissue shape and actual ventricular laminar architecture, which likely caps its functional output and leaves electromechanical performance short of adult levels [22]. Some platforms remain limited by baseline electrophysiological variability such as differences in conduction and action potential upstroke velocities across different stem cell lines, which complicates cross-donor comparisons [23,99]. The device uses a micro-spherical stress gauge to measure tissue-scale contractile forces through pillar deflection and local cell-scale stresses through the optical deformation of embedded hydrogel sensors. However, the deformation and stress calculations were inaccurate, as compressive strains were either underestimated or unaccounted for [18]. Some heart chips (e.g., The SpheroFlow) are limited by inadequate imaging quality for immunofluorescence microscopy and weak pacing electrodes, which use a prolonged pulse width that may hinder optimal cardiac maturation and metabolic readout accuracy [19,100,101].

### 4.6. Lack of Drug Diversity

In drug testing, the primary limitation of this bioprinted platform is the tendency of PDMS to absorb small drug molecules, which restricts its utility for accurate drug testing [39]. This could be solved through the use of modified polymer surfaces or biomaterials such as quantum dot-doped thermoplastic elastomers. The heart model’s limitation in drug testing is its evaluation using only one drug type (verapamil), failing to capture diverse pharmacological responses such as drug-induced oxidative stress or electrical signal suppression [72]. Similarly, some platforms relied on only two drugs (isoproterenol and nifedipine) that target heart rate, failing to evaluate broader pathological mechanisms like oxidative stress or electrical signal suppression [75]. Rather than only using one or two drug types, other drugs such as doxorubicin and flecainide, which play different pathological roles, can be used [72,102].

### 4.7. The Potential of HoC in Regenerative Medicine

Although HoC itself cannot directly regenerate damaged heart tissue, it may contribute to the development and testing of regenerative treatments before future clinical applications [52,103]. Specifically, HoC platforms offer transformative potential in regenerative medicine by serving as biomimetic testing grounds for cell- and tissue-based therapies. By leveraging patient-derived stem cells, these microfluidic devices enable personalized disease modeling, precision screening of mRNA or gene-editing vectors, and pre-clinical validation of functional cardiomyocyte maturation [103]. Furthermore, these systems allow researchers to evaluate candidate biomaterials and engineered scaffolds under physiological shear stress and mechanical strain, dramatically accelerating the development of targeted, patient-specific cardiac repair strategies.

## 5. Conclusions

We reviewed a number of studies on HoC platforms and identified multiple technological advances in their development. Microfluidics, human stem cell-derived cardiac tissue, tissue architecture and biomaterial scaffolds, and mechanical and electrical stimulation have been integrated to fabricate a variety of modern HoC platforms. The replication of cardiac structure and function in these systems has greatly improved, promoting cardiomyocyte development. These platforms demonstrate considerable potential to be used as precise, physiologically relevant cardiac models for cardiovascular research, such as disease modeling and drug testing. Despite these advancements, these platforms are still limited by low reproducibility, immature stem cells, insufficient vascularization, limited cell diversity, incomplete replication of the cardiac microenvironment and its function, and a lack of drug diversity in existing research. Continued improvements in these areas can further enhance these HoC platforms, making them valuable, clinically translatable tools for cardiovascular research.

## Figures and Tables

**Table 1:** Key parameters and applications in the various heart chips.

No.	Cell Type	Culture Type	Fab & Methods	Mechanical Stimulation	Electrical Stimulation	Cell Alignment/Synchronized HBR	Applications	Ref.
1	NRCMs, hiPSC-CMs, hFBs	Short-term, Dyn-3D-Co	PDMS replica-molding, 3D bioprinting, Micro-electrode coaxial guide tech	Dir: Uniaxial A: 10% strain F: 1 Hz D: 7 days	A: 1–5 V	CA: Yes Syn HBR: 35 bpm	Drug screening (sotalol, verapamil, aspirin), electrophysiology	[12]
2	hiPS-CMs	Longterm, Dyn, mono	PDMS stamp, Resin anchors w/pillar, PDMS cantilever,	N/A	A: 1.2 V F: N/A D: 20 ms	CA: Yes Syn HBR: ~0.59 Hz	Heart modeling	[13]
3	iPSC-CMs, hvcFBs, EPIs-CMs	Longterm, Static-3D Tri	Biowire II, TPE/QD microwires; soft lithography, hydrogel	N/A	A: 1.00 V F: 1–2 Hz D: 2 ms	CA: Yes Syn HBR: Yes, NS	IRI modeling, Epicardial-myocardial crosstalk modeling	[14]
4	HUVECs, NRCMs, hiPSC-CMs	Long-term, Dyn-3D-Co	2-layer PDMS sandwiched between PMMA frames. Bioprinting	N/A	N/A	CA: Yes Syn HBR: NRCMs: 50–70 bpm for 2 weeks. hiPSC-CMs: ~60 bpm for 10 days.	Drug screening (doxorubicin)	[15]
5	hiPSC-CMs, cFBs, ECs	Short-term, Dyn-3D-Tri	CNC milling, Heart chip with PTFE	Natural contraction	D: 7 days	CA: Yes Syn HBR: spontaneous	Drug testing (isoproterenol, doxorubicin)	[16]
6	NRCMs, hiPSC-CMs, hFBs, hECs	Short-term, Dyn-3D-Co	PDMS replica-molding 3D bioprinting Micro-electrode coaxial guide	N/A	A: Increased by 0.5 V until ET F: 2 Hz D: 7 days	CA: No Syn HBR: Spontaneous	Drug screening (verapamil) Electrophysiology, mechanobiology	[17]
7	NRVCMs, cFBs	Long-term, Dyn-3D-Co	PDMS replica molding, two-phase oil-in-water emulsion	Dir: Uniaxial	D: 14 days	CA: Yes Syn HBR: Yes, 25–65 bpm	Disease model, drug screening, mechanobiology	[18]
8	hiPSC-CM, EC, MC	Long-term, Dyn-3D-Co	PDMS replica-molding	N/A	N/A	CA: Yes Syn HBR: Yes, 50–70 bpm	Heart model, drug testing (isoproterenol)	[2]
9	hiPSC-CMs, hFBs	Long-term, Dyn-3D-Co	PDMS stamp micromolding, PET membrane	N/A	A: 5 V F: 1 Hz D: Long-term pacing	CA: Yes Syn HBR: Yes, 60 bpm	Heart model, drug testing	[19]
10	hiPSC-CMs, hcmECs, hvFBs	Short-term, Dyn-3D-Tri	PDMS replica-molding 3D bioprinting.	N/A	N/A	CA: Yes Syn HBR: Yes, 50–60 bpm	Heart disease modeling, drug testing	[20]
11	hiPSC-CMs, NRVCMs	Short-term, Dyn-3D-mono	PDMS replica-molding Microcontact printing	N/A	A: 5 V F: 1 Hz D: Short-term pacing	CA: Yes Syn HBR: Yes, 60 bpm	Disease modeling (Barth syndrome), drug testing.	[21]
12	hiPSC-CMs, hFBs	Long-term, Dyn-3D-Co	CNC milling elastomeric pillars, hydrogel scaffold, Carbon-rod electrodes	N/A	A: 5 V F: 2 Hz D: ~4 weeks	CA: Yes Syn HBR: Yes, 120 bpm	Disease modeling, drug testing	[22]
13	hiPSC-CMs, hESC-CMs, iCell, hcFBs	Long-term, Dyn-3D-Co	PDMS replica-molding, POMaC polymer wires, hydrogel	N/A	A: 50–90 mV F: 0.4–6 Hz D: 7 days	CA: Yes Syn HBR: Yes, NS	Heart modeling, drug testing	[23]
14	hiPSC-CMs	Long-term, Static-3D-Mono	PDMS replica-molding	N/A	A: 5–10 V F: 1–2 Hz D: 10 ms	CA: Yes Syn HBR: Yes, NS	Heart modeling, drug testing	[24]
15	hiPSC-CMs, ECs	Long-term, Dyn-3D-Co	Microfluidic-chip	N/A	A: ±1000 μV F: 0.5 Hz D: 1 ms	CA: Yes Syn HBR: ~39 bpm	Personalized medicine, drug testing, heart modeling	[25]
16	NRVCMs	Long-term, Static-3D-Mono	Direct 3D bioprinting	N/A	A: 70 V F: 0.5–4 Hz D: 10 ms	CA: yes Syn HBR: yes, NS	Disease modeling, drug screening	[26]
17	ESCs	Short-term, Static-3D-Mono	Microfluidic chip	N/A	N/A	CA: No Syn HBR: 60 bpm	Heart modeling, drug screening/testing	[27]
18	hiPSC-CMs	Long-term, Dyn-3D-Co	Microfluidic chip	A: 10% deformation F: 0.4 Hz D: 7 days	N/A	CA: Yes Syn HBR: 60 bpm	Heart modeling, drug screening/testing	[28]
19	NRVCMs	Short-term, Static-2D-Mono	PDMS chip with microcontact printing	N/A	F: 1–2 Hz	CA: No Syn HBR: 120 bpm	Disease modeling, drug testing	[29]
20	Cardiac SMCs	Short-term, Dyn-2D-Mono	Heart-chip	Dir: Uniaxial A: 10% strain F: 3 Hz D: 96 h	A: 2–5 V F: 2 Hz D: ≥5 min	CA: Yes Sync HBR: N/A	Failing myocardium model, drug testing	[30]
21	NRVCMs	Long-term, Dyn-3D-Mono	3D printing, soft lithography	F: 0.4 Hz D: 3–9 days	F: 1, 2, 3 Hz	CA: Yes Syn HBR: Yes, NS	Heart disease modeling, drug testing	[31]
22	hiPSC-CMs	Long-term, Dyn-3D-Mono	Heart-chip	N/A	A: 0–10 V F: 1–10 Hz D: 4 ms	CA: Yes Syn HBR: N/A	Heart modeling, drug testing	[32]
23	hCMs, hFBs	Long-term, Dyn-3D-Co	Heart-chip	Dir: Uniaxial A: 9% strain F: 1 Hz D: 96 h	N/A	CA: Yes Syn HBR: N/A	Heart disease modeling, drug testing	[33]
24	Human vascular, liver, heart, bone	Long-term, Dyn-3D-Co	Microfluidic chip	F: 2–6 Hz D: 4 weeks	A: 4.5 V F: 2–6 Hz D: 2 ms	CA: Yes Syn HBR: N/A	Multi-organ modeling, disease modeling, drug testing	[34]
25	CMs	Long-term, Dyn-3D-Mono	Microgrooved chip with electrical stimulation	N/A	A: ±2 V F: 3.3 Hz D: 1 ms	CA: Yes Syn HBR: NS	Heart modeling, drug screening.	[35]
26	hiPSC-CMs	Short-term, Dyn-3D-Co	Heart chip	Natural contraction	N/A	CA: Yes Syn HBR: No, spontaneous	Heart modeling	[36]
27	hiPSC-CMs, ECs, FBs	Short-term, Dyn-3D-Co	Heart chip	Natural contraction	N/A	CA: N/A Syn HBR: No, spontaneous	Drug testing	[37]
28	hiPSC-CMs	Short-term, Dyn-2D-Mono	Microvalve-based bioprinting device	Natural contraction	Natural electrical activity	CA: N/A Syn HBR: No, spontaneous	Drug testing	[8]
29	NRVCMs	Short-term, Static-3D-Mono	Heart chip/Nanofibers	Dir: Uniaxial F: 0.5, 1, 2, 3 Hz	A: 10 V F: 1, 2, 3 Hz	CA: Yes Syn HBR: No, spontaneous	Heart modeling	[38]
30	hiPSC-CMs, cFBs	Long-term, Static-3D-Co	Heart chip/electrical stimulation	Natural contraction	A: 2× ET F: 1–2 Hz D: 4 weeks	CA: N/A Syn HBR: No, spontaneous	Drug testing	[39]
31	iPSC-CMs, iPSC-FBs	Short-term, Dyn-3D-Co	PDMS molding, 3D wax printing, electrical sensors	N/A	F: 3.2–4.2 HZ D: 5 h	CA: No Syn HBR: N/A	Therapeutic screening of cardiac fibrosis, mechanistic studies	[40]
32	Human pericytes, cECs, cFBs, CMs	Long-term, Dyn-3D-Co	Photo-/soft lithography	N/A	No	CA: Yes Syn HBR: No, spontaneous	Drug testing/development	[41]
33	hepatocytes,hiPSC-CMs	Short-term, Dyn-3D-Co	Photolithography, Microelectrode arrays	N/A	A: 0.6–1 V F: 0.5–4 Hz D: 1 ms bipolar pulse	CA: N/A Syn HBR: No, spontaneous	Drug development	[42]
34	hiPSC-CMs	Long-term, Dyn-3D-Mono	Photolithography, PDMS	N/A	N/A	CA: Yes Syn HBR: Yes	Drug testing (nanoparticles)	[43]
35	H9c2, HepG2	Short-term, Dyn-3D-Co	Photolithography	N/A	N/A	CA: N/A Syn HBR: Yes	Drug testing	[44]
36	RVCMs	Short-term, 2D-Mono	Photo-/soft lithography, microcontact printing	D: 12 h	N/A	CA: Yes Syn HBR: No	Heart modeling	[45]
37	hiPSC-CM, hvcBs	Short-term, Static-3D-Co	Heart chip/PDMS, PMMA	N/A	A: 1 V F: 1 Hz	CA: No Syn HBR: No	Drug testing	[46]
38	hiPSC-CMs, HUVEC, blood cells	Long-term, Dyn-3D-Co	Photo-/soft lithography, 3D stamping	N/A	N/A	CA: Yes Syn HBR: Yes	Drug screening, mechanistic studies	[47]
39	NRCMs, H9c2, Nor10	Short-term, Dyn-3D-Co	Photolithography	N/A	N/A	CA: Yes Syn HBR: Yes	Heart modeling	[48]
40	hiPSC-CMs, ECs, SMCs, hcFBs	Long-term, Dyn-3D-Co	PDMS replica-molding/PMMA	N/A	N/A	CA: Yes Syn HBR: Yes	Heart modeling	[49]
41	hiPSC-CMs	Long-term, Dyn-3D-Co	Microfluidic chip, hydrogel scaffolds	Dir: Uniaxial	A: 2x ET F: 2–6 Hz D: 3 weeks	CA: Yes Syn HBR: 1–6 Hz	Disease modeling, drug screening	[50]
42	hiPSC-CMs	Long-term, Dyn-3D-Co	Microfluidic PDMS chip with wires	F: 0.5 Hz D: 1 h/day for 5 days	N/A	CA: Yes Syn HBR: N/A	Disease modeling, drug testing	[51]
43	hiPSC-CMs	Long-term, Dyn-3D-Co	Microfluidic chip	N/A	F: 1 Hz D: 14 days	CA: Yes Syn HBR: 1 Hz	Vascularization modeling	[52]
44	hiPSC-CMs, EPIs	Long-term, Dyn-3D-Co	Biowire, micro-post, or scaffold-guided alignment.	N/A	A: 1 V F: 1 Hz D: 3–7 days	CA: Yes Syn HBR: 1 Hz	Disease modeling (e.g., IRI)	[53]
45	hiPSC-CMs, hiSC-EC, hiSC-FBs	Short-term, Dyn-3D-Co	Microfluidic chip	N/A	F: 0.8–1 Hz	CA: Yes Syn HBR: rhythmic spontaneous	Disease (inflammation) modeling, drug screening	[54]
46	hiPSC-CMs	Long-term, Dyn-3D-Mono	Photo-/soft lithography, microelectrodes	N/A	A: 3–4 V/cm F: 1–6 Hz D: 26 days	CA: Yes Syn HBR: Yes, 61.5 bpm	Disease/physiology modeling, drug screening/testing	[55]
47	NRCMs	Mono	CNT conductive ink, Photolithography, PDMS stamps	N/A	A: Increased by 0.5 V F: 22.85 Hz D: 10 days	CA: No Syn HBR: arrhythmic	Heart modeling, drug testing/screening	[56]
48	hPSC-CMs, ECs, hcFBs	Long-term, Dyn-3D-Co	Microfluidic PDMS chip	N/A	N/A	CA: Yes Syn HBR: Yes	Disease modeling, drug testing/screening	[57]
49	hiPSC-CMs, hCFs	Long-term, Static-3D-Co	Photo-/soft lithography	N/A	D: 14 days	CA: Yes Syn HBR: Yes	Disease modeling (e.g., IRI)	[11]
50	NRCMs	Long-term, Dyn-2D-Mono	PDMS replica molding	N/A	F: 2 Hz	CA: Yes Syn HBR: arrhythmic	Heart modeling, drug screening	[9]
51	hiPSC-CMs	Long-term, Dyn-3D-Mono	PMMA-PET membrane chip	N/A	F: 20 Hz D: 7 days	CA: Yes Syn HBR: 21.6 bpm	Disease modeling, drug testing	[58]

Note: Human induced pluripotent stem cell-derived cardiomyocytes (hiPSC-CMs), human cardiac microvascular endothelial cells (hcmECs), neonatal rat ventricular cardiomyocyte (NRVCMs), ischemia–reperfusion injury (IRI), epicardial cells (EPIs), human pericytes, cardiac endothelial cells (hECs), cardiac fibroblasts (cFBs), cardiac myocytes (CMs), hepatocellular carcinoma cells (HepG2), embryonic stem cell-derived cardiac spheroids (ESC), smooth muscle cells (SMCs), human umbilical vein endothelial cells (HUVECs), amplitude (A), frequency (F), duration (D), cell alignment (CA), synchronized HBR (syn HBR), stretching direction (SD), heart beat rate (HBR), not specified (NS), static culture (S), dynamic culture (Dyn).

## Data Availability

No new data were created or analyzed in this study. Data sharing is not applicable to this article.
